# Looking at the carcinogenicity of human insulin analogues via the intrinsic disorder prism

**DOI:** 10.1038/srep23320

**Published:** 2016-03-17

**Authors:** Elrashdy M. Redwan, Moustafa H. Linjawi, Vladimir N. Uversky

**Affiliations:** 1 Department of Biological Sciences, Faculty of Sciences, King Abdulaziz University, P.O. Box 80203, Jeddah 21589, Saudi Arabia; 2 Therapeutic and Protective Proteins Laboratory, Protein Research Department, Genetic Engineering and Biotechnology Research Institute, City for Scientific Research and Technology Applications, New Borg EL-Arab 21934, Alexandria, Egypt; 3 Department of Laboratory Technology, Faculty of Applied Medical Sciences, King Abdulaziz University, P.O. Box 80203, Jeddah 21589, Saudi Arabia; 4 Department of Molecular Medicine and USF Health Byrd Alzheimer’s Research Institute, Morsani College of Medicine, University of South Florida, Tampa, FL, USA; 5 Laboratory of Structural Dynamics, Stability and Folding of Proteins, Institute of Cytology, Russian Academy of Sciences, St. Petersburg, Russian Federation, Russia

## Abstract

Therapeutic insulin, in its native and biosynthetic forms as well as several currently available insulin analogues, continues to be the protein of most interest to researchers. From the time of its discovery to the development of modern insulin analogues, this important therapeutic protein has passed through several stages and product generations. Beside the well-known link between diabetes and cancer risk, the currently used therapeutic insulin analogues raised serious concerns due to their potential roles in cancer initiation and/or progression. It is possible that structural variations in some of the insulin analogues are responsible for the appearance of new oncogenic species with high binding affinity to the insulin-like growth factor 1 (IGF1) receptor. The question we are trying to answer in this work is: are there any specific features of the distribution of intrinsic disorder propensity within the amino acid sequences of insulin analogues that may provide an explanation for the carcinogenicity of the altered insulin protein?

Insulin, a 51 amino acid 5.7 kDa protein comprised of an A and B chain linked by two disulphide bridges and produced by pancreatic islet β cells, is one of the best-known and most-studied proteins^1^. The pioneering work of Stanley Cohen and Herbert Boyer, who invented the technique of DNA cloning, signaled the birth of genetic engineering, which allowed genes to be transferred between different biological species with ease^2^. This discovery led to the development of a realm of recombinant proteins with various therapeutic applications, such as insulin and growth hormone. Genes encoding human insulin and growth hormone were cloned and expressed in *E. coli* in 1978 and 1979 respectively^3^. The first licensed drug produced using recombinant DNA technology was human insulin, which was developed by Genentech and licensed as well as marketed by Eli Lilly in 1982^4^,^5^. Recombinant production of human insulin was a real breakthrough because it opened a possibility for diabetes treatment at mass with human protein, an obvious step forward. Until this discovery, diabetic patients had been treated with non-human insulins, which were purified from bovine or porcine pancreas using a technique established in the early 1920s^6^.

More recently, insulin analogues have been engineered to enhance desired molecular properties (e.g. rapid absorption or prolonged duration of action) without altering immunogenicity^2^. These improvements have enabled physicians to tailor treatment regimens and more closely emulate the normal insulin physiology comprising a stable basal secretion with surges of insulin which are temporally closely due to food ingestion.

The incidence of diabetes is increasing at an alarming rate, and it has been speculated that the number of diabetic patients worldwide will increase to approximately 300 million by the year 2025^7^. Consequently, the requirement for insulin as a therapeutic will increase manifold (approximately to more than 16,000 kg/year), and the current productivity of existing insulin expression systems would not be sufficient to meet future market demand. Therefore, efficient expression systems for insulin production are urgently needed, and novel routes for insulin administration such as oral or inhalation also have to be developed^8^.

Amino acid sequences of animal insulin from different mammals are rather similar to the human insulin sequence. For example, porcine insulin has only a single amino acid difference from the human protein, whereas the bovine insulin differs by three amino acids. Both porcine and bovine insulins are active in humans and interact with human insulin receptor with approximately the same strength as their human analogue. These two proteins are widely used in isophane neutral protamine hagedorn (isophane NPH) formulation, which is the stoichiometric mixture of insulin and protamine at neutral pH, to subcutaneous injection, frequently utilized for basal insulin support in diabetes type 1 and type 2. Before biosynthesis of human insulin using DNA technology became available, bovine and porcine insulin were the first commercially available and clinically used insulin preparations. They were not the only proteins used in the diabetes treatment, however, as the shark insulin was widely used in Japan^6^. Unfortunately, in some patients, the non-human insulins have caused allergic reactions mostly related to the extent of purification. Although the formation of non-neutralizing antibodies is rarely observed with recombinant human insulin, an allergy may still occur in some patients due to the preservatives used in insulin preparations. Currently, biosynthetic insulin is broadly distributed worldwide and largely replaced the animal insulin^4^,^6^.

Before biosynthetic human recombinant analogues were available, semisynthetic insulins were clinically used based on the specific chemical modifications of animal insulins. For example, Novo Nordisk enzymatically converted porcine insulin into semisynthetic ‘human’ insulin by removing the single amino acid that varies from the human variety, and chemically adding the human amino acid. Chemical modifications of the amino acid side chains at the N-terminus and/or the C-terminus were made in order to alter the absorption, distribution, metabolism, and excretion (ADME) characteristics of the insulin analogues^6^. Since self-association of insulin into large aggregates represents significant mechanical problems in insulin delivery devices and might cause complications during diabetes treatment, chemical modifications of this protein were elaborated to generate insulin analogues that can resist aggregation. Although one of these modifications, sulfated insulin (SI), has been known for some time, clinical usage of this analogue has been limited due to the major insulin bioactivity losses that occurred during SI production^9^. However, an approach was developed in 1983 that could generate the highly active, non-aggregating sulfated insulin, with the controlled sulfation degree varying over an eightfold range^9^.

Normal unmodified insulin is soluble at physiological pH. Analogues have been created that have a shifted isoelectric point so that they are engaged in a solubility equilibrium where the majority of the protein first precipitates amorphously, and is then slowly released to the bloodstream according to zero-order kinetics, and is eventually excreted by the kidneys. These insulin analogues are used to replace the basal level of insulin, and may be effective over a period of up to 24 hours. One of these slow-acting insulin analogues, insulin Detemir, binds to albumin via fatty acid chain, thereby providing slow absorption and a prolonged metabolic effect^10^. However, results from long-term usage of such analogues (e.g. more than 10 years), which are obviously required for assessment of clinical benefit, are currently not available.

Unmodified human and porcine insulins tend to complex with zinc in the blood and form stable hexamers. Insulin in the hexameric form cannot bind to its receptors. Therefore, the hexamer has to dissociate into monomers for insulin to be biologically active. At physiological conditions, such dissociation is a slow process. As a result, hexameric insulin delivered subcutaneously is not readily available for the body when insulin is needed in larger doses, such as after a meal (although this is more a function of subcutaneously administered insulin, as intravenously dosed insulin is distributed rapidly to the cell receptors, and therefore does not have this problem). Zinc-complexed insulin is used for slow release of basal insulin. Basal insulin support is required throughout the day, representing about 50% of daily insulin requirement^2^, whereas the insulin amount needed at mealtime constitutes the remaining 50%. Non-hexameric insulins (monomeric insulins) were developed to be faster-acting agents and to replace the injection of normal unmodified insulin before a meal. There are phylogenetic examples for such monomeric insulins in animals^11^.

## Biological Links Between Diabetes and Cancer Risks

Carcinogenesis and malignant transformation is a complex process that can be divided into multiple steps: initiation, promotion, and progression. Factors that affect one or more steps of this pathway might be associated with cancer incidence or mortality. Possible mechanisms for a direct link between diabetes and cancer include hyperinsulinemia, hyperglycemia, or chronic inflammation^12^,^13^,^14^. Hyperinsulinemia may be endogenous because of insulin resistance, or exogenous due to the administered insulin or insulin secretagogues. It was also pointed out that there is a potential link between diabetes therapies and cancer^15^,^16^,^17^,^18^,^19^.

Furthermore, insulin and the insulin-like growth factor (IGF) axis have a number of effects on cancer cells. Human tumors commonly over-express insulin receptors (IR) and IGF1 receptors^20^. Insulin receptor may be expressed in two different isoforms, IR-A and IR-B, differing by 12 amino acids due to the alternative splicing of exon 11^21^. In malignant cells, expression of the IR-A isoform lacking exon 11 is predominant^22^. This form of the IR possesses high IGF2 affinity, which is close to that of insulin. Interaction of IR-A with insulin is known to lead primarily to metabolic effects, whereas activation of IR-A by IGF2 results primarily in mitogenic effects^22^. In general, contrary to IR-B, activation of IR-A elicits more mitogenic than metabolic effects^22^. Therefore, insulin may favor cancer progression and facilitate the growth of tumors^23^.

When both the IR and IGF1 receptors interact with their ligands, multiple signaling pathways are activated, which leads to phosphorylation of adaptor proteins such as the insulin receptor substrate family. These signaling pathways may promote proliferation, protection from apoptotic stimuli, invasion, and metastasis, potentially enhancing promotion and progression of many types of cancer^24^. Elevated levels of insulin have been shown to be a risk factor for a number of cancers. This conclusion was confirmed by meta-analysis^25^, which showed the excess risks of colorectal, pancreatic, and breast cancers in patients with high insulin levels.

Apart from direct effects of insulin on cancer cells, it is possible that hyperinsulinemia could promote carcinogenesis indirectly through the effects of insulin effects on IGF1^26^. High insulin levels have been shown to stimulate IGF1 in animals, which is known to increase the risk of colorectal cancer^27^. As mentioned earlier, cancer cells over-express IRs and IGF1 receptors. Insulin reduces the hepatic production of insulin-like growth-factor-binding protein-1 (IGFBP-1)^28^ and IGFBP-2^29^. This leads to the increased levels of circulating free, bioactive IGF1. In turn, this could act as a growth stimulus in preneoplastic and neoplastic cells that express receptors of these proteins^30^. It is important to note here that IGF1 has more potent mitogenic and anti-apoptotic activities than insulin^31^.

People with circulating IGF1 have an increased risk of common epithelial cancers such as breast, colon, and prostate^32^,^33^,^34^,^35^,^36^,^37^,^38^. Prospective studies performed by The Endogenous Hormones and Breast Cancer Collaborative Group^39^ have shown that women with the highest concentration of IGF1 have a 28% higher risk of developing breast cancer than women with the lowest concentration. In breast cancer, insulin induces P450 aromatase activity and reduces sex hormones binding globulin (SHBG); these increased levels of free oestrogen in turn increase mitogenicity^12^,^40^. A meta-analysis of 43 prospective and cross-sectional studies indicated lower levels of SHBG and higher levels of oestrogen and testosterone amongst patients with type 2 diabetes, compared with controls, even after adjustment for obesity^41^,^42^. Elevated endogenous sex steroid levels are associated with a higher risk of postmenopausal breast and endometrial cancers, and possibly other cancer types^43^,^44^.

Although hyperinsulinemia may suppress prostate carcinogenesis by reducing levels of active IGF1, a putative risk factor for prostate cancer^45^ some other explanations are possible. The reduction in risk of prostate cancer has been suggested to be related to lower levels of testosterone in men with diabetes^46^, as higher androgens levels are known to be associated with an increased risk of prostate cancer^47^,^48^. Tumor cells exhibit enhanced glucose metabolism compared to normal tissue. The altered metabolism of cancer cells characterized by high rates of glucose consumption and glycolysis was described by Otto Warburg 81 years ago^12^,^49^. Most diabetic patients present both hyperglycemia and hyperinsulinemia. Thus, it is difficult to distinguish the specific role of hyperglycemia in the increased cancer risks. Neoplastic cells use glucose for proliferation, and one of the central characteristics of malignant tissues is increased metabolism of glucose towards the pentose phosphate pathway^12^,^50^. Therefore, a higher circulating glucose concentration may foster cancer development by providing an amiable environment for the growth of malignant cell clones. Theories of cancer energetics focus on the role of glycolysis to generate adenosine triphosphate, which in turn fuels the high-energy requirements of tumor growth. The recent resurgence of interest in the Warburg hypothesis and cancer energetics emphasizes the dependence of many cancers on glycolysis for energy, creating a high requirement for glucose, because ATP generation by glycolysis requires far more glucose than oxidative phosphorylation.

Hypoxia is one of the hallmarks of cancer. The presence of hypoxia has been demonstrated in different types of solid tumors. Thus, it has been hypothesized that hyperglycemia drives mitogenic activity. However, evidence suggests that this is not the case as most cancers have highly effective upregulated, insulin-independent glucose uptake mechanisms and therefore may not derive a further growth advantage from hyperglycemia. Beyond glucotoxic generation of reactive species and DNA damage, hyperglycemia may supplement the effect of hyperinsulinemia^51^. Glucose deprivation may induce oxidative stress and glucotoxicity. Therefore, further evidence suggests a role for the oxidative stress-responsive genes that are sensitive to hyperglycemia and regulate the level of reactive oxygen species (ROS)^12^,^52^.

The metabolic abnormalities that characterize diabetes mellitus increase oxidative stress and cause permanent pro-inflammatory conditions. This state reduces intracellular anti-oxidant capacity, predisposing susceptible cells to malignant transformation. High concentrations of free radicals and oxidants generate a potent ROS that can damage cell DNA, causing mutations. ROS may also react with other cellular molecules such as proteins and lipids, forming derivative products. These products may alter intracellular homeostasis that predispose for accumulation of mutations that contribute to the carcinogenesis process^12^,^53^.

## Insulin Analogues Carcinogenicity

The IGF1 receptor binding has been linked to tumor development in rodents^23^,^54^, and these findings have led to the discontinuation of several specific insulin analogues. Currently available insulin analogues exhibit an affinity for the IGF1 receptor ranging between 16 and ~650% relative to native human insulin, depending on the specific insulin analogue and cell line studied^55^,^56^,^57^. The speed of insulin dissociation from the IR may also contribute to the mitogenic potential of insulin analogues^55^.

Insulin is required for all patients with type 1 diabetes, and is also necessary for many patients with type 2 diabetes to treat hyperglycemia. Insulin and IGF1 and their receptors and their intracellular signaling pathways share large similarities. Likewise, the metabolic and mitogenic effects of the two hormones partially overlap^12^,^18^. Both insulin and insulin analogues, by stimulating the insulin and IGF1 receptors, can function as growth factors stimulating mitogenesis^55^. Several formulations of insulin exist: short-acting human regular insulin, intermediate-acting human neutral protamine Hagedorn insulin, and both rapid-acting and long-acting analogues of human insulin^58^. It was demonstrated that modification of the molecular structure of insulin could result in increased mitogenic properties in cell lines and animal models^12^,^18^.

There have been concerns related to the mitogenic activity and the potential for carcinogenicity of one of the insulin analogues, Glargine^59^. Several epidemiological studies have been performed to address these issues. Recently, the results of the 6.5 year Origin study with Glargine have been published^60^,^61^. Also, a series of widely publicized epidemiologic analyses examined a possible association between insulin use and/or use of long-acting insulin analogue Glargine and cancer^16^,^17^,^62^,^63^. Insulin Glargine may have a disparate impact on cancer risk through its elevated binding to IGF1 receptors^12^,^64^,^65^. There have been four large retrospective observational studies looking into a possible causal link^18^. The study examined the potential oncogenic effects of human insulin and several insulin analogues, such as glargine, aspart, and lispro. The analysis found a dose-dependent increased risk of malignancy with insulin Glargine compared to human insulin^12^,^66^ and also a higher rate for breast cancer in patients receiving insulin Glargine monotherapy relative to those on insulin glargine combined with other insulin preparations^12^,^67^. On the other hand, randomized clinical trial data from a 5-year trial of insulin Glargine versus neutral protamine Hagedorn insulin did not provide evidence of enhanced cancer risk in the insulin Glargine arm^12^,^68^. Also, other observations showed no evidence of increased cancer risk in patients receiving insulin Glargine relative to insulin-naïve patients^12^,^69^,^70^. There was no evidence of an increased risk of cancer associated with insulin Detemir, either^12^,^70^,^71^.

However, it is clear that all insulin analogs must be tested for carcinogenicity, as insulin can be engaged in cross-talk with IGF receptor pathways, which can cause abnormal cell growth and tumorigenesis. The modifications to insulin always carry the risk of unintentionally enhancing IGF signaling in addition to the desired pharmacological properties^12^,^18^.

## Some Structural Features and Disorder Propensity of Human Insulin and IGFs

Insulin and IGFs belong to the insulin superfamily, whose members are synthesized as prepro-proteins consisting of 4 domains (pre, B, C, A). These are then processed by proteolytic removal of the pre-domain. In human insulin (UniProt ID: P01308, 110 residues), in addition to the proteolytic removal of pre-domain (or signal peptide, residues 1–24), processing involves post-translational proteolytic removal of the C-domain (or propeptide, residues 57–87), eventually generating the well-folded mature protein, in which the A and B domains (residues 90–110 and 25–54, respectively) are covalently linked by two disulfide bonds (Cys_31_-Cys_96_ and Cys_43_-Cys_109_). Another disulfide bond (Cys_95_-Cys_100_) provides additional stability to the chain A.

The IGF precursors have additional C-terminal propeptides, which are also removed post-translationally by specific proteolysis. Therefore, the precursor of human IGF1 (UniProt ID: P05019, 195 residues) contains a signaling peptide (residues 1–21), a propeptide (residues 22–48), an IGF1 domain (residues 49–118), and a C-terminal propeptide (or E-peptide, residues 119–195). There are three disulfide bonds in the single-chain mature IGF1, Cys_54_-Cys_96_, Cys_66_-Cys_109_, and Cys_95_-Cys_100_. The precursor of human IGF2 (UniProt ID: P01344, 180 residues) has slightly different domain organization and contains a signaling peptide (residues 1–24), an IGF2 domain (residues 25–91) and a C-terminal propeptide (or E-peptide, residues 92–180) that also includes a preptin domain (residues 92–126). Similar to insulin and IGF1, the mature IGF2 possesses three disulfide bonds, Cys_33_-Cys_71_, Cys_45_-Cys_84_, and Cys_70_-Cys_75_. In other words, the cystein residues 1, 2, 3, 4, 5, and 6 in all these proteins are cross-linked to form an identical disulfide pattern 1–4, 2–6, and 3–5.

**Figure 1:**
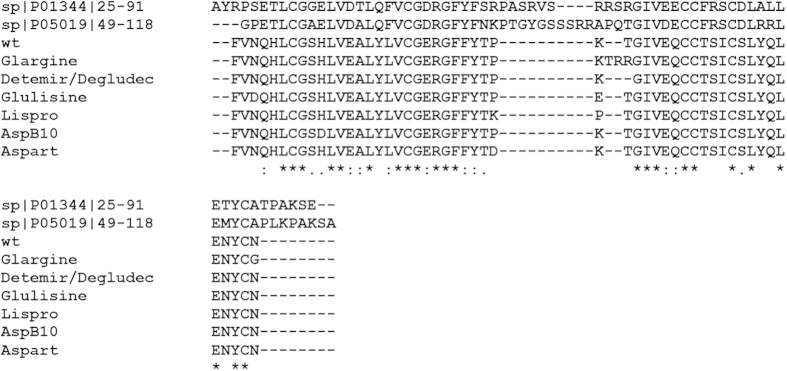
Multiple sequence alignments of mature forms of human IGF1, IGF2, insulin and insulin analogues.

**Figure 2:**
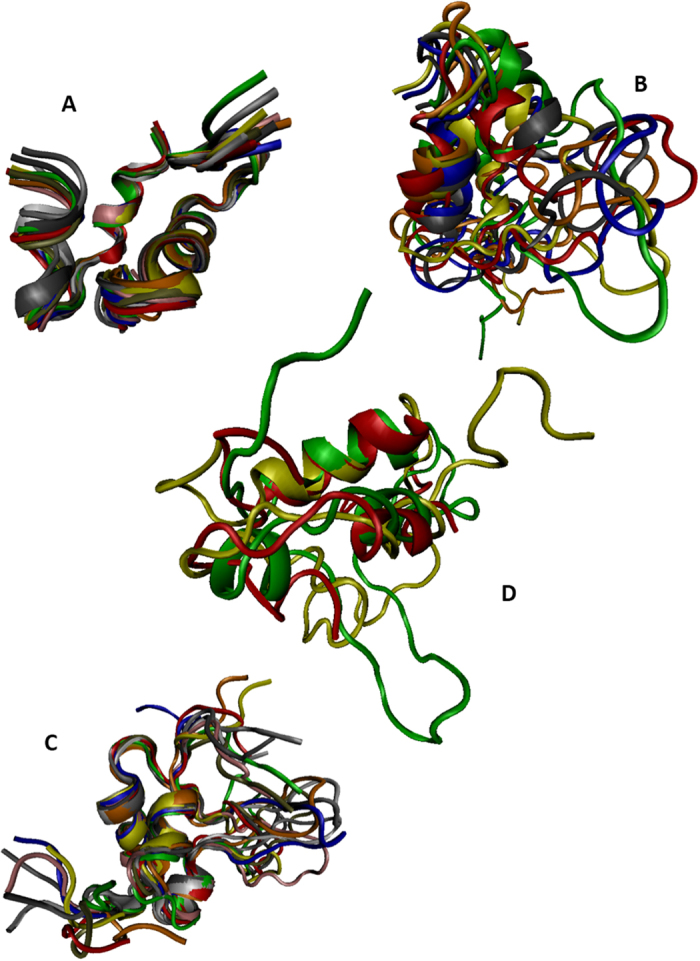
Solution structures of human insulin (**A**) PDB ID: 1a7f), human IGF1 (**B**) PDB ID: 1bqt), and human IGF2 (**C**) PDB ID: 1igl). Central image (**D**) represents results of structural alignment of human IGF1 (yellow structure, PDB ID: 1bqt), IGF2 (green structure; PDB ID: 1igl) and insulin (red structure, PDB ID: 1a7f).

Figure 1 shows that, in addition to similar disulfide patterns, mature human IGFs, human insulin, and insulin analogues have high sequence similarity. Therefore, it is not surprising that these proteins, together with other members of the insulin superfamily, are characterized by a very similar basic fold. This conclusion is illustrated by Fig. 2 which represents NMR solution structures of human IGF1, IGF2, and insulin and their structural alignment. It has been emphasized that, although the lengths of the secondary structural elements in known structures of the members of the insulin superfamily might vary, and although the connecting loops can be highly flexible and likely to appear as dynamic ensembles of interconverting conformations (or be absent, as in insulin), the insulin-like fold can be divided to two (sub)domains, A and B. Single long α-helix of the (sub)domain B covers 2 shorter α-helices of the (sub)domain A.

**Figure 3:**
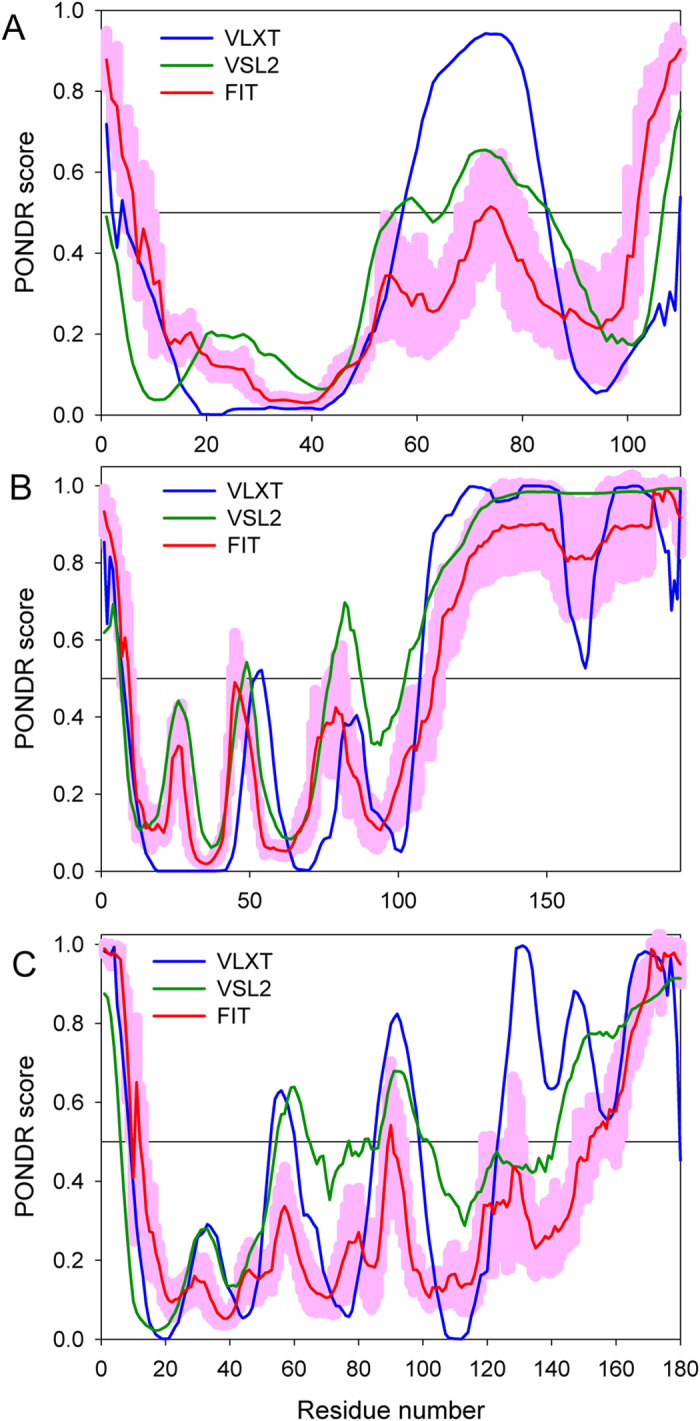
Per residue intrinsic disorder propensity of pre-proforms of human insulin (**A**) UniProt ID: P01308), IGF1 (**B**) UniProt ID: P05019), and IGF2 (**C**) UniProt ID: P01344). Disorder predispositions were evaluated by PONDR^®^ VLXT (blue line)^72^, PONDR^®^ VSL2 (green line)^110^, and PONDR-FIT (red line)^75^. The light pink shadow around PONDR-FIT curves represents the distribution of the statistical error of PONDR-FIT predictions. In these plots, residues and regions with scores above 0.5 are considered as disordered.

Even simple visual analysis of Fig. 2 indicates that solution structures of human insulin and IGFs possess rather different dynamic properties, with insulin being the least flexible and IGF1 being the most dynamic molecule. To understand the predisposition of amino acid sequences of these proteins for intrinsic disorder we analyzed the pre-propolypeptides and the mature forms of human insulin, IGF1, and IGF2 by the members of the PONDR family, such as PONDR^®^ VLXT^72^, PONDR^®^ VSL2^73^, PONDR^®^ VL3^74^, and PONDR^®^ FIT^75^. In these analyses, the predicted intrinsic disorder scores (PIDSs) above 0.5 are considered to correspond to the disordered residues/regions, whereas regions with a disorder score 0.2 < PIDS < 0.5 are considered flexible. PONDR^®^ VSL2 is one of the more accurate stand-alone disorder predictors^73^,^76^,^77^. PONDR^®^ VLXT has high sensitivity to local sequence peculiarities associated with disorder-based interaction sites^72^. PONDR^®^ VL3 is one of the more accurate evaluators of long disordered regions^74^, whereas PONDR-FIT, being a metapredictor combining six individual predictors (PONDR^®^ VLXT^78^, PONDR^®^ VSL2^73^, PONDR^®^ VL3^74^, FoldIndex^79^, IUPred^80^, and TopIDP^81^) is moderately more accurate than each of the component predictors^75^. Results of this analysis are shown in Fig. 3. According to this PONDR-based analysis, pre-proproteins are predicted to have significant amounts of intrinsic disorder, with pre-pro-IGF1 expected to be the most disordered of these three proteins. Curiously, sites attacked by proteases during the IGF1, IGF2, and insulin maturations are located either within the disordered (PIDS >0.5) or flexible (PIDS >0.2) regions of these proteins. In fact, in pre-proinsulin, significant parts of the signal peptide (residues 1–24) and the propeptide (or the C-domain, residues 57–87) are predicted to be disordered. One can observe similar tendencies in the pre-proIGF1 and pre-proIGF2, where the signal peptides (residues 1–21 and 1–24) in the precursors of human IGF1 and IGF2, propeptide (residues 22–48) of the IGF1, and the C-terminal propeptides (or E-peptides, residues 119–195 and 92–180) in the precursors of human IGF1 and IGF2 are all predicted to be mostly disordered.

These correlations between predicted disorder and functional sites of proteins are in line with recent findings which suggest that the functionality of many proteins depends on intrinsic disorder, that functions of such disordered proteins complement functions of ordered proteins and domains^72^,^82^,^83^,^84^,^85^,^86^,^87^,^88^,^89^,^90^,^91^,^92^,^93^, and that intrinsically disordered proteins (IDPs) and hybrid proteins with ordered domains and functional disordered regions are very common in any given proteome^72^,^91^,^94^,^95^,^96^. Often, IDPs play crucial roles in regulation and control of various signaling pathways^86^,^97^. Furthermore, structure, conformational stability and biological functions of IDPs are commonly modulated and controlled by various posttranslational modifications (PTMs), such as phosphorylation, acetylation, lipidation, ubiquitination, sumoylation, glycosylation, proteolytic cleavage, etc., with sites of these PTMs often located within disordered regions^83^,^98^,^99^,^100^,^101^.

## Increased Intrinsic Disorder Propensity in Some Insulin Analogues as a Marker of their Increased Mitogenicity

**Figure 4:**
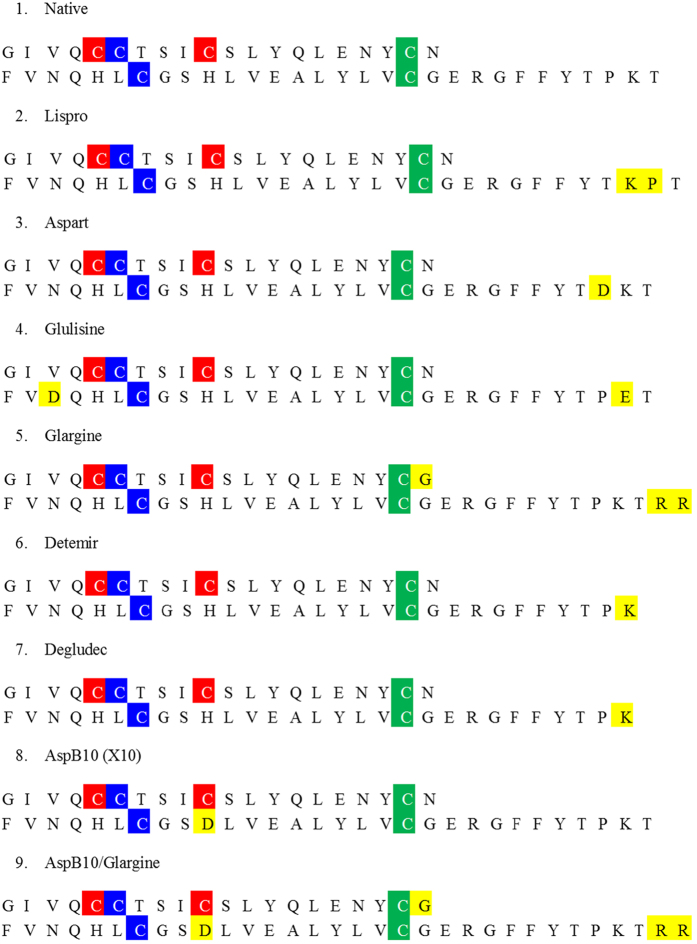
Amino acid sequences of insulin analogues. Upper peptide is A-chain, lower peptide is B-chain. Cysteines involved in the formation of disulfide bonds are shown as red, blue and green boxes. Yellow boxes show where the modification was entered into the native recombinant human insulin to generate specific insulin analogues.

Genetic engineering of DNA was used to change the amino acid sequence of natural insulin to produce insulin analogues or IR binding agonists with altered physiological properties, such as absorption, distribution, metabolism, and excretion characteristics of insulin^102^. Figure 4 shows amino acid sequences of the insulin analogues analyzed below. For insulin Lispro, proline at position 28 and lysine at position 29 in the B-chain of human insulin are interchanged. For insulin Aspart, the proline at position 28 in the B-chain is replaced by aspartic acid. For insulin Glulisine, the asparagine at position 3 and lysine at position 29 in the B-chain are replaced by lysine and glutamic acid, respectively. For insulin Glargine, aspartic acid at position 21 in the A-chain had been replaced for glycine and the B-chain contains two extra amino acids (two arginines) at positions 31 and 32. For insulin Detemir, threonine at position 30 of the B-chain is removed and a 14-carbon fatty acid chain (myristic acid) is added to position 29 of the B-chain. Similarly, for insulin Degludec, threonine at position 30 of the B-chain is removed and the hexadecanedioic acid is conjugated at K (B29). Finally, AspB10 insulin has a His-Asp substitution at position 10 of the B-chain. These modifications of native insulin were needed mostly to affect the dissociation rate of zinc-bound insulin hexamers (which is a natural form of this protein that is not able to interact with IR) to biologically active monomers. As a result, modifications used in the Glulisine, Aspart, and Lispro were introduced to generate short-acting insulin analogues (i.e., analogues that dissociate more rapidly than the native insulin following injection), whereas modifications used in Glargine, Detemir, and Degludec were made to produce long-acting insulin analogues (i.e., insulins with delayed absorption or a prolonged duration of action)^102^. Table 1 lists approved generations of insulin analogues.

It was pointed out that, although substitutions at the C-terminus of the insulin B-chain (the B26–B30 region) which were used to generate many of the insulin analogues practically do not affect insulin binding to the IR, some of the insulin analogues with mutated residues in the B-chain are characterized by increased structural homology with IGF1 and also have an enhanced affinity to the IGF1 receptor^55^,^102^,^103^. For example, when the proline residue at position B28 is substituted with basic residues, the relative affinity of the resulting insulin analogues to IGF1 receptor increases ∼1.5- to 2-fold^103^. Compared to human insulin, the affinity of Glargine (which has extra arginine residues at position B31–32) for the IGF1 receptor is increased tenfold, and it can further increase to 28-fold by the aspartic acid substitution at B10 of the Glargine^102^. It was pointed out that the insulin analogues with the substantially increased affinity for the IGF1 receptors might possess an increased potency to stimulate proliferation of cells^102^. Because many of the primary tumors and malignant cells are characterized by an increased expression of IGF1 receptors, the aforementioned higher affinities of some insulin analogues to these receptors could be of clinical importance^23^.

The question then arose if intrinsic disorder predisposition can be used to evaluate mitogenicity of insulin analogues. To answer this question, we compared the peculiarities of the disorder profiles (distributions of the per-residue intrinsic disorder propensities within the sequences of query proteins). Direct analysis of the predisposition of insulins for intrinsic disorder is complicated by the fact that in their mature forms, insulin and its analogues contain two short polypeptide chains crosslinked by disulfide bonds. These A- and B-chains are too short for reliable evaluation of intrinsic disorder by the majority of currently available computational tools that typically have a length threshold of 30 residues. Furthermore, it is likely that the aforementioned topological organization of the mature insulins might overcome intrinsic predisposition of their short A- and B-chains for disorder. To avoid these complications, we ignored the disulfide bonds, and instead of looking at short chains we examined the artificial constructs where, for a given insulin or its analogue, the C-terminus of a B-chain was directly linked to the N-terminus of the corresponding A-chain. In this way, we were able to see how different mutations introduced in insulin analogues affect the predisposition of entire polypeptide chain for intrinsic disorder. The amino acid sequences of these artificial constructs of human insulin and its analogues can be reliably aligned with the sequences of human IGF1 and IGF2 (see Fig. 1).

**Figure 5:**
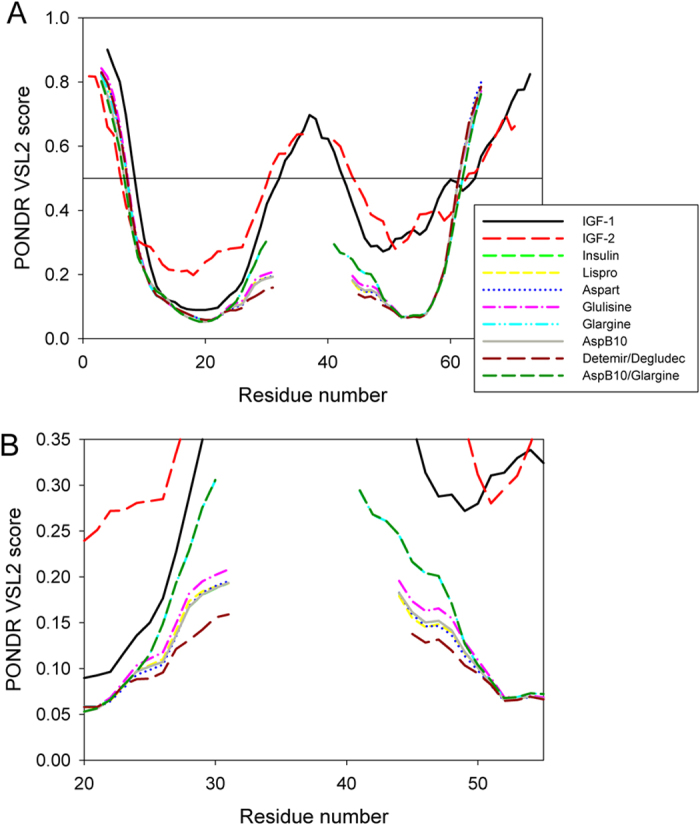
Intrinsic disorder propensities of aligned mature human IGF1, IGF2, insulin, and insulin analogues evaluated by PONDR^®^ VSL2. Plot (**A**) is a full-scale representation of the corresponding disorder profiles, whereas plot (**B**) shows the zoomed part of the plot corresponding to the central region of these proteins.

Figure 5 compares intrinsic disorder propensities of the aligned IGF1 and IGF2 with those of insulin and its therapeutic analogues. It is seen that nature IGF1 and IGF2 are predicted to have disordered tails and a central IDPR. Although artificial constructs of insulins corresponding to the computationally linked B- and A-chains are predicted to be more ordered than IGF1 and IGF2, they also are expected to have disordered tails and a central region with increased flexibility. Figure 5 also shows that the termini of these artificial insulin constructs are not affected by the introduced modifications, whereas the flexibility of this central region increases in the following order: Detemir/Degludec < AspB10 = insulin = Aspart = Lispro < Glulisin < Glargin = AspB10/Glargin. Curiously, Glargin and its modified form, AspB10/Glargin, which are known to have the highest affinity to the IGF1 receptor *in vitro* among all insulin analogues^102^, are characterized by the highest levels of intrinsic disorder/flexibility in their central regions. This observation suggests that the analysis of disorder profiles might have some predictive power for evaluation of the mitogenicity/carcinogenicity of insulin analogues. Therefore, the use of the corresponding computational tools for sequence-based evaluation of intrinsic disorder predisposition is recommended while developing new insulin analogues.

It is of interest to compare this utilization of intrinsic disorder knowledge with that used for the design of engineered disordered tags^104^,^105^. In fact, it has been emphasized that the most commonly used tags are short (typically being around 20 residues at maximum) disordered tails that can mediate highly specific and diverse functions, ranging from serving as tunable affinity probes to act as covalent coupling points^104^. Longer disordered tails (so-called entropic bristle domains, EBDs) can be used as protein solubility enhancers^106^. Such EBDs extend away from the protein to which they are fused and sweep out large molecules, thereby allowing the target protein to fold free from interference^106^. These EBDs can be rationally designed as sequences with low levels of sequence complexity and a high net charge, and are easily diversified by means of using distinctive amino acid compositions and lengths^106^. When elastin-like peptides (ELPs) (which are specific peptides derived from the elastin and characterized by the ability to undergo a reversible phase transition from the structurally disordered, highly solvated conformation below the inverse transition temperature (*T*_*t*_) to a new phase comprising desolvated and aggregated polypeptides when the temperature is raised above *T*_*t*_) are used as tags, the means is generated for the chromatography-free purification of target proteins^104^,^105^,^107^,^108^,^109^. Here, the principle of the “inverse transition cycling” (ITC) is utilized, where the ELP fusion protein is first rendered insoluble in aqueous solution by triggering its inverse transition. At the next stages, this aggregated ELP fusion protein is first collected by centrifugation, and then resolubilized in buffer at a temperature below the *T*_*t*_ to yield a soluble target protein^107^.

## Methods

In this work, we evaluated the per residue intrinsic disorder predispositions of pre-proforms of human insulin (UniProt ID: P01308), IGF1 (UniProt ID: P05019), and IGF2 (UniProt ID: P01344) by a set of predictors from the PONDR family, such as PONDR^®^ VLXT^72^, PONDR^®^ VSL2^110^, and PONDR-FIT^75^. Per-residue disorder propensity of human insulin analogues was studied by PONDR^®^ VSL2^110^. The choice of these tools is defined by their specific peculiarities. Although PONDR^®^ VLXT is not the most accurate predictor, this computational tool has high sensitivity to local sequence peculiarities which are often associated with disorder-based interaction sites^72^, PONDR^®^ VSL2 is one of the most accurate stand-alone disorder predictors^110^, whereas PONDR-FIT represents a metapredictor which is moderately more accurate than each of the component predictors, is one of the most accurate disorder predictors^75^.

## Additional Information

**How to cite this article**: Redwan, E. M. *et al.* Looking at the carcinogenicity of human insulin analogues via the intrinsic disorder prism. *Sci. Rep.* **6**, 23320; doi: 10.1038/srep23320 (2016).

## Figures and Tables

**Table 1:** Approved generations of insulin products (native, recombinant, analogues).

Generation	Description	Mode of action	Subtype	No. of amino acid	Source	Brand name	Nonproteinous modification	Expasy characteristic pI/Mw
First	It is extracted from animals, undergoes a chemical modification to be functional insulin with intermediate duration of action. It is crystalline zinc suspension mixed with positively charged protamine	Normal	One	51 a.a.	Extraction and purification from animals	Humulin N, Novolin N, Novolin NPH, Gensulin N, SciLin N, NPHIletin II, Isophane	None	6.02/5666.53
Second	Human insulin gene sequences are used to produce insulin by DNA technology using different expression system. Final active insulin are identical to native insulin protein	Normal	One	51 a.a.	Biosynthetic (recombination) in Yeast or *E.coli*	Humulin, Insuman, Novolin, Actrapid, Velosunlin, Monotrad, Insultrad, Protaphane, Mistrad, Actraphone, Ultratard	None	6.02/5666.53
Third	Based on desire to have short- or long-acting insulin, specific modifications were introduced into the 2^nd^ generation molecule. These goals were achieved, but modifications subsequently altered the original form and/or structure of insulin thereby generating insulin analogues.	Short acting	Lispro	51 a.a.	Biosynthetic (recombination) in Yeast or *E.coli*	Humalog, Liprolog,	None	6.02/5666.53
			Aspart	51 a.a.		Novolog, Novorapid		5.36/5684.51
			Glulisine	51 a.a.		Apidea		4.56/5668.46
		Long acting	Detemir	50 a.a	Biosynthetic (recombination) in Yeast or *E.coli*	Levemir	Myristic acid conjugation at B29	6.02/5793.801
			Degludec	50 a.a.		Tresiba	Hexadecanoecioic acid conjugation at B29	6.02/5821.854
			Glargine	53 a.a.		Lantus, Otisulin, Toujeo, Abasaglor, Basaglar	Non	7.78/5921.86

## References

[b1] Redwan R. M., Matar S. M., El-Aziz G. A. & Serour E. A. Synthesis of the human insulin gene: protein expression, scaling up and bioactivity. Prep Biochem Biotechnol 38(1), 24–39 (2008). 18080908 10.1080/10826060701774312

[b2] Owens D. R. Insulin preparations with prolonged effect. Diabetes Technol Ther 13 Suppl 1, S5–14 (2011). 21668337 10.1089/dia.2011.0068

[b3] Granner D. K. Hormones of the pancreas and GI tract in Haper’s Biochemistry, edited by Murray R. K., Mayes P. A., Granner D. K., & Rodwell V. W. (Appleton & Lange, California, 1990), pp. 530–545.

[b4] Redwan R. M. Cumulative updating of approved biopharmaceuticals. Hum Antibodies 16 **(3–4)**, 137–158 (2007). 18334749

[b5] Walsh G. Biopharmacetiucal approval trends in 2009. BioPharm Int. 23(10), 30–41 (2010).

[b6] Redwan R. M. Animal-derived pharmaceutical proteins. J Immunoassay Immunochem 30(3), 262–290 (2009). 19591041 10.1080/15321810903084400

[b7] Wild S., Roglic G., Green A., Sicree R. & King H. Global prevalence of diabetes: estimates for the year 2000 and projections for 2030. Diabetes Care 27(5), 1047–1053 (2004). 15111519 10.2337/diacare.27.5.1047

[b8] Baeshen N. A. *et al.* Cell factories for insulin production. Microb Cell Fact 13, 141, 10.1186/s12934-014-0141-0 (2014). 25270715 PMC4203937

[b9] Pongor S., Brownlee M. & Cerami A. Preparation of high-potency, non-aggregating insulins using a novel sulfation procedure. Diabetes 32(12), 1087–1091 (1983). 6360757 10.2337/diab.32.12.1087

[b10] Philips J. C. & Scheen A. Insulin detemir in the treatment of type 1 and type 2 diabetes. Vasc Health Risk Manag 2(3), 277–283 (2006). 17326333 10.2147/vhrm.2006.2.3.277 PMC1993987

[b11] Horuk R. *et al.* A monomeric insulin from the porcupine (Hystrix cristata), an Old World hystricomorph. Nature 286(5775), 822–824 (1980). 6995860 10.1038/286822a0

[b12] Szablewski L. Diabetes mellitus: influences on cancer risk. Diabetes Metab Res Rev 30(7), 543–553 (2014). 25044584 10.1002/dmrr.2573

[b13] Suh S. & Kim K. W. Diabetes and cancer: is diabetes causally related to cancer? Diabetes Metab J 35(3), 193–198 (2011). 21785737 10.4093/dmj.2011.35.3.193 PMC3138100

[b14] Vigneri P., Frasca F., Sciacca L., Pandini G. & Vigneri R. Diabetes and cancer. Endocr Relat Cancer 16(4), 1103–1123 (2009). 19620249 10.1677/ERC-09-0087

[b15] Li C. & Kong D. Cancer risks from diabetes therapies: evaluating the evidence. Pharmacol Ther 144(1), 71–81 (2014). 24844968 10.1016/j.pharmthera.2014.05.006

[b16] Hsieh M. C. *et al.* The influence of type 2 diabetes and glucose-lowering therapies on cancer risk in the Taiwanese. Exp Diabetes Res 2012, 413782; 10.1155/2012/413782 (2012). 22719752 PMC3374948

[b17] Currie C. J., Poole C. D. & Gale E. A. The influence of glucose-lowering therapies on cancer risk in type 2 diabetes. Diabetologia 52(9), 1766–1777 (2009). 19572116 10.1007/s00125-009-1440-6

[b18] Seewoodhary J. & Bain S. C. Diabetes, diabetes therapies and cancer: what’s the link? Br J Diabetes Vasc Dis 11(5), 235–238 (2011).

[b19] Vigneri R. Diabetes: diabetes therapy and cancer risk. Nat Rev Endocrinol 5(12), 651–652 (2009). 19924151 10.1038/nrendo.2009.219

[b20] Papa V. *et al.* Elevated insulin receptor content in human breast cancer. J Clin Invest 86(5), 1503–1510 (1990). 2243127 10.1172/JCI114868 PMC296896

[b21] Moller D. E., Yokota A., Caro J. F. & Flier J. S. Tissue-specific expression of two alternatively spliced insulin receptor mRNAs in man. Mol Endocrinol 3(8), 1263–1269 (1989). 2779582 10.1210/mend-3-8-1263

[b22] Frasca F. *et al.* Insulin receptor isoform A, a newly recognized, high-affinity insulin-like growth factor II receptor in fetal and cancer cells. Mol Cell Biol 19(5), 3278–3288 (1999). 10207053 10.1128/mcb.19.5.3278 PMC84122

[b23] Pollak M. Insulin and insulin-like growth factor signalling in neoplasia. Nat Rev Cancer 8(12), 915–928 (2008). 19029956 10.1038/nrc2536

[b24] Mardilovich K., Pankratz S. L. & Shaw L. M. Expression and function of the insulin receptor substrate proteins in cancer. Cell Commun Signal 7, 14 (2009). 19534786 10.1186/1478-811X-7-14 PMC2709114

[b25] Pisani P. Hyper-insulinaemia and cancer, meta-analyses of epidemiological studies. Arch Physiol Biochem 114(1), 63–70 (2008). 18465360 10.1080/13813450801954451

[b26] Giovannucci E. Insulin, insulin-like growth factors and colon cancer: a review of the evidence. J Nutr 131(11) Suppl, 3109S–3120S (2001). 11694656 10.1093/jn/131.11.3109S

[b27] Volkers N. Diabetes and cancer: scientists search for a possible link. J Natl Cancer Inst 92(3), 192–194 (2000). 10655433 10.1093/jnci/92.3.192

[b28] Ooi G. T., Tseng L. Y., Tran M. Q. & Rechler M. M. Insulin rapidly decreases insulin-like growth factor-binding protein-1 gene transcription in streptozotocin-diabetic rats. Mol Endocrinol 6(12), 2219–2228 (1992). 1283442 10.1210/mend.6.12.1283442

[b29] Renehan A. G., Frystyk J. & Flyvbjerg A. Obesity and cancer risk: the role of the insulin-IGF axis. Trends Endocrinol Metab 17(8), 328–336 (2006). 16956771 10.1016/j.tem.2006.08.006

[b30] Werner H. & Le Roith D. New concepts in regulation and function of the insulin-like growth factors: implications for understanding normal growth and neoplasia. Cell Mol Life Sci 57(6), 932–942 (2000). 10950308 10.1007/PL00000735 PMC11146963

[b31] Weinstein D., Simon M., Yehezkel E., Laron Z. & Werner H. Insulin analogues display IGF-I-like mitogenic and anti-apoptotic activities in cultured cancer cells. Diabetes Metab Res Rev 25(1), 41–49 (2009). 19145584 10.1002/dmrr.912

[b32] Pollack M. N. Insulin, insulin-like growth factors, insulin resistance, and neoplasia. Am J Clin Nutr 86(3), s820–822 (2007). 18265475 10.1093/ajcn/86.3.820S

[b33] Pollak M. Insulin, insulin-like growth factors and neoplasia. Best Pract Res Clin Endocrinol Metab 22(4), 625–638 (2008). 18971123 10.1016/j.beem.2008.08.004

[b34] Pollak M. N. Insulin-like growth factors and neoplasia. Novartis Found Symp 262, 84–98, discussion 98–107, 265–108 (2004). 15562824

[b35] Pollak M. N., Schernhammer E. S. & Hankinson S. E. Insulin-like growth factors and neoplasia. Nat Rev Cancer 4(7), 505–518 (2004). 15229476 10.1038/nrc1387

[b36] Schairer C. *et al.* Circulating insulin-like growth factor (IGF)-I and IGF binding protein (IGFBP)-3 levels and postmenopausal breast cancer risk in the prostate, lung, colorectal, and ovarian cancer screening trial (PLCO) cohort. Horm Cancer 1(2), 100–111 (2010). 21761353 10.1007/s12672-010-0013-y PMC10358053

[b37] Eng-Wong J. *et al.* Premenopausal breast cancer: estrogen receptor status and insulin-like growth factor-I (IGF-I), insulin-like growth factor binding protein-3 (IGFBP-3), and leptin. Breast J 15(4), 426–428 (2009). 19601950 10.1111/j.1524-4741.2009.00753.x

[b38] Tas F., Karabulut S., Bilgin E., Tastekin D. & Duranyildiz D. Clinical significance of serum insulin-like growth factor-1 (IGF-1) and insulin-like growth factor binding protein-3 (IGFBP-3) in patients with breast cancer. Tumour Biol 35(9), 9303–9309 (2014). 24943688 10.1007/s13277-014-2224-2

[b39] Endogenous H. *et al.* Insulin-like growth factor 1 (IGF1), IGF binding protein 3 (IGFBP3), and breast cancer risk: pooled individual data analysis of 17 prospective studies. Lancet Oncol 11(6), 530–542 (2010). 20472501 10.1016/S1470-2045(10)70095-4 PMC3113287

[b40] McTernan P. G. *et al.* Gender differences in the regulation of P450 aromatase expression and activity in human adipose tissue. Int J Obes Relat Metab Disord 24(7), 875–881 (2000). 10918534 10.1038/sj.ijo.0801254

[b41] Ding E. L., Song Y., Malik V. S. & Liu S. Sex differences of endogenous sex hormones and risk of type 2 diabetes: a systematic review and meta-analysis. JAMA 295(11), 1288–1299 (2006). 16537739 10.1001/jama.295.11.1288

[b42] Wolf I. & Rubinek T. Diabetes mellitus and breast cancer In Diabetes and Cancer. Epidemiological Evidence and Molecular Links. Frontiers Diabetes. (eds Masur K., Thévenood F. & Zänker K. S. ) 97–113 (Karger, 2008).

[b43] Allen N. E. *et al.* Endogenous sex hormones and endometrial cancer risk in women in the European Prospective Investigation into Cancer and Nutrition (EPIC). Endocr Relat Cancer 15(2), 485–497 (2008). 18509001 10.1677/ERC-07-0064 PMC2396334

[b44] Giovannucci E. *et al.* Diabetes and cancer: a consensus report. Diabetes Care 33(7), 1674–1685 (2010). 20587728 10.2337/dc10-0666 PMC2890380

[b45] Renehan A. G. *et al.* Insulin-like growth factor (IGF)-I, IGF binding protein-3, and cancer risk: systematic review and meta-regression analysis. Lancet 363(9418), 1346–1353 (2004). 15110491 10.1016/S0140-6736(04)16044-3

[b46] Giovannucci E. & Michaud D. The role of obesity and related metabolic disturbances in cancers of the colon, prostate, and pancreas. Gastroenterology 132(6), 2208–2225 (2007). 17498513 10.1053/j.gastro.2007.03.050

[b47] Wolk A., Andersson S. O. & Bergstrom R. Prospective study of sex hormone levels and risk of prostate cancer. J Natl Cancer Inst 89(11), 820; 10.1093/jnci/89.11.820 (1997). 9182984

[b48] Gann P. H., Hennekens C. H., Ma J., Longcope C. & Stampfer M. J. Prospective study of sex hormone levels and risk of prostate cancer. J Natl Cancer Inst 88(16), 1118–1126 (1996). 8757191 10.1093/jnci/88.16.1118

[b49] Warburg O. The Metabolism of Tumors. (Smith, New York, 1931).

[b50] Dang C. V. & Semenza G. L. Oncogenic alterations of metabolism. Trends Biochem Sci 24(2), 68–72 (1999). 10098401 10.1016/s0968-0004(98)01344-9

[b51] Yun J. *et al.* Glucose deprivation contributes to the development of KRAS pathway mutations in tumor cells. Science 325(5947), 1555–1559 (2009). 19661383 10.1126/science.1174229 PMC2820374

[b52] Turturro F., Friday E. & Welbourne T. Hyperglycemia regulates thioredoxin-ROS activity through induction of thioredoxin-interacting protein (TXNIP) in metastatic breast cancer-derived cells MDA-MB-231. BMC Cancer 7, 96, 10.1186/1471-2407-7-96 (2007). 17555594 PMC1906826

[b53] Ohshima H., Tatemichi M. & Sawa T. Chemical basis of inflammation-induced carcinogenesis. Arch Biochem Biophys 417(1), 3–11 (2003). 12921773 10.1016/s0003-9861(03)00283-2

[b54] Pollak M. The insulin and insulin-like growth factor receptor family in neoplasia: an update. Nat Rev Cancer 12(3), 159–169 (2012). 22337149 10.1038/nrc3215

[b55] Kurtzhals P. *et al.* Correlations of receptor binding and metabolic and mitogenic potencies of insulin analogs designed for clinical use. Diabetes 49(6), 999–1005 (2000). 10866053 10.2337/diabetes.49.6.999

[b56] Sommerfeld M. R. *et al.* *In vitro* metabolic and mitogenic signaling of insulin glargine and its metabolites. PLoS One 5(3), e9540; 10.1371/journal.pone.0009540 (2010). 20209060 PMC2832019

[b57] Ciaraldi T. P., Carter L., Seipke G., Mudaliar S. & Henry R. R. Effects of the long-acting insulin analog insulin glargine on cultured human skeletal muscle cells: comparisons to insulin and IGF-I. J Clin Endocrinol Metab 86(12), 5838–5847 (2001). 11739448 10.1210/jcem.86.12.8110

[b58] Szablewski L. Therapies and emerging targets for the treatment of diabetes In Glucose Homeostasis and Insulin Resistance (ed Szablewski L. ) 175–204 (Bentham, 2011).

[b59] Varewijck A. J., Yki-Jarvinen H., Schmidt R., Tennagels N. & Janssen J. A. Concentrations of insulin glargine and its metabolites during long-term insulin therapy in type 2 diabetic patients and comparison of effects of insulin glargine, its metabolites, IGF-I, and human insulin on insulin and igf-I receptor signaling. Diabetes 62(7), 2539–2544 (2013). 23569175 10.2337/db12-1773 PMC3712030

[b60] Gerstein H. C. *et al.* Basal insulin and cardiovascular and other outcomes in dysglycemia. N Engl J Med. 367(4), 319–328 (2012). 22686416 10.1056/NEJMoa1203858

[b61] Hanefeld M. & Bramlage P. Insulin use early in the course of type 2 diabetes mellitus: the ORIGIN trial. Curr Diab Rep. 13(3), 342–349 (2013). 23397557 10.1007/s11892-013-0366-z PMC3647081

[b62] Butler P. C. Insulin glargine controversy: a tribute to the editorial team at Diabetologia. Diabetes 58(11), 2427–2428 (2009). 19875618 10.2337/db09-9030 PMC2768168

[b63] Currie C. J. The longest ever randomised controlled trial of insulin glargine: study design and HbA(1c) findings. Diabetologia 52(10), 2234–2235; author reply 2236-2239 (2009). 19662377 10.1007/s00125-009-1477-6

[b64] Gerstein H. C. Does insulin therapy promote, reduce, or have a neutral effect on cancers? JAMA 303(5), 446–447 (2010). 20124540 10.1001/jama.2010.60

[b65] Smith U. & Gale E. A. Does diabetes therapy influence the risk of cancer? Diabetologia 52(9), 1699–1708 (2009). 19597799 10.1007/s00125-009-1441-5

[b66] Hemkens L. G. *et al.* Risk of malignancies in patients with diabetes treated with human insulin or insulin analogues: a cohort study. Diabetologia 52(9), 1732–1744 (2009). 19565214 10.1007/s00125-009-1418-4 PMC2723679

[b67] Jonasson J. M. *et al.* Insulin glargine use and short-term incidence of malignancies-a population-based follow-up study in Sweden. Diabetologia 52(9), 1745–1754 (2009). 19588120 10.1007/s00125-009-1444-2

[b68] Rosenstock J. *et al.* Similar risk of malignancy with insulin glargine and neutral protamine Hagedorn (NPH) insulin in patients with type 2 diabetes: findings from a 5 year randomised, open-label study. Diabetologia 52(9), 1971–1973 (2009). 19609501 10.1007/s00125-009-1452-2 PMC2723677

[b69] Colhoun H. M. & Group S. E. Use of insulin glargine and cancer incidence in Scotland: a study from the Scottish Diabetes Research Network Epidemiology Group. Diabetologia 52(9), 1755–1765 (2009). 19603149 10.1007/s00125-009-1453-1 PMC2723678

[b70] Tennagels N. & Werner U. The metabolic and mitogenic properties of basal insulin analogues. Arch Physiol Biochem 119(1), 1–14 (2013). 23373726 10.3109/13813455.2012.754474 PMC3581051

[b71] Dejgaard A., Lynggaard H., Rastam J. & Krogsgaard Thomsen M. No evidence of increased risk of malignancies in patients with diabetes treated with insulin detemir: a meta-analysis. Diabetologia 52(12), 2507–2512 (2009). 19838665 10.1007/s00125-009-1568-4

[b72] Dunker A. K. *et al.* Intrinsically disordered protein. J Mol Graph Model 19(1), 26–59 (2001). 11381529 10.1016/s1093-3263(00)00138-8

[b73] Peng K. *et al.* Optimizing long intrinsic disorder predictors with protein evolutionary information. J Bioinform Comput Biol 3(1), 35–60 (2005). 15751111 10.1142/s0219720005000886

[b74] Peng K., Radivojac P., Vucetic S., Dunker A. K. & Obradovic Z. Length-dependent prediction of protein intrinsic disorder. BMC Bioinformatics 7, 208; 10.1186/1471-2105-7-208 (2006). 16618368 PMC1479845

[b75] Xue B., Dunbrack R. L., Williams R. W., Dunker A. K. & Uversky V. N. PONDR-FIT: a meta-predictor of intrinsically disordered amino acids. Biochim Biophys Acta 1804(4), 996–1010 (2010). 20100603 10.1016/j.bbapap.2010.01.011 PMC2882806

[b76] Peng Z. L. & Kurgan L. Comprehensive comparative assessment of in-silico predictors of disordered regions. Curr Protein Pept Sci 13(1), 6–18 (2012). 22044149 10.2174/138920312799277938

[b77] Fan X. & Kurgan L. Accurate prediction of disorder in protein chains with a comprehensive and empirically designed consensus. J Biomol Struct Dyn 32(3), 448–464 (2014). 23534882 10.1080/07391102.2013.775969

[b78] Romero P. *et al.* Sequence complexity of disordered protein. Proteins 42(1), 38–48 (2001). 11093259 10.1002/1097-0134(20010101)42:1<38::aid-prot50>3.0.co;2-3

[b79] Prilusky J. *et al.* FoldIndex: a simple tool to predict whether a given protein sequence is intrinsically unfolded. Bioinformatics 21(16), 3435–3438 (2005). 15955783 10.1093/bioinformatics/bti537

[b80] Dosztanyi Z., Csizmok V., Tompa P. & Simon I. IUPred: web server for the prediction of intrinsically unstructured regions of proteins based on estimated energy content. Bioinformatics 21(16), 3433–3434 (2005). 15955779 10.1093/bioinformatics/bti541

[b81] Campen A. *et al.* TOP-IDP-scale: a new amino acid scale measuring propensity for intrinsic disorder. Protein Pept Lett 15(9), 956–963 (2008). 18991772 10.2174/092986608785849164 PMC2676888

[b82] Wright P. E. & Dyson H. J. Intrinsically unstructured proteins: re-assessing the protein structure-function paradigm. J Mol Biol 293(2), 321–331 (1999). 10550212 10.1006/jmbi.1999.3110

[b83] Dunker A. K., Brown C. J., Lawson J. D., Iakoucheva L. M. & Obradovic Z. Intrinsic disorder and protein function. Biochemistry 41(21), 6573–6582 (2002). 12022860 10.1021/bi012159+

[b84] Tompa P. Intrinsically unstructured proteins. Trends Biochem Sci 27(10), 527–533 (2002). 12368089 10.1016/s0968-0004(02)02169-2

[b85] Dyson H. J. & Wright P. E. Intrinsically unstructured proteins and their functions. Nat Rev Mol Cell Biol 6(3), 197–208 (2005). 15738986 10.1038/nrm1589

[b86] Uversky V. N., Oldfield C. J. & Dunker A. K. Showing your ID: intrinsic disorder as an ID for recognition, regulation and cell signaling. J Mol Recognit 18(5), 343–384 (2005). 16094605 10.1002/jmr.747

[b87] Dunker A. K., Cortese M. S., Romero P., Iakoucheva L. M. & Uversky V. N. Flexible nets. The roles of intrinsic disorder in protein interaction networks. FEBS J 272(20), 5129–5148 (2005). 16218947 10.1111/j.1742-4658.2005.04948.x

[b88] Xie H. *et al.* Functional anthology of intrinsic disorder. 1. Biological processes and functions of proteins with long disordered regions. J Proteome Res 6(5), 1882–1898 (2007). 17391014 10.1021/pr060392u PMC2543138

[b89] Vucetic S. *et al.* Functional anthology of intrinsic disorder. 2. Cellular components, domains, technical terms, developmental processes, and coding sequence diversities correlated with long disordered regions. J Proteome Res 6(5), 1899–1916 (2007). 17391015 10.1021/pr060393m PMC2588346

[b90] Xie H. *et al.* Functional anthology of intrinsic disorder. 3. Ligands, post-translational modifications, and diseases associated with intrinsically disordered proteins. J Proteome Res 6(5), 1917–1932 (2007). 17391016 10.1021/pr060394e PMC2588348

[b91] Uversky V. N. & Dunker A. K. Understanding protein non-folding. Biochim Biophys Acta 1804(6), 1231–1264 (2010). 20117254 10.1016/j.bbapap.2010.01.017 PMC2882790

[b92] Tompa P. Intrinsically disordered proteins: a 10-year recap. Trends Biochem Sci. 37(12), 509–516 (2012). 22989858 10.1016/j.tibs.2012.08.004

[b93] Uversky V. N. A decade and a half of protein intrinsic disorder: biology still waits for physics. Protein Sci 22(6), 693–724 (2013). 23553817 10.1002/pro.2261 PMC3690711

[b94] Dunker A. K., Obradovic Z., Romero P., Garner E. C. & Brown C. J. Intrinsic protein disorder in complete genomes. Genome Inform Ser Workshop Genome Inform 11, 161–171 (2000). 11700597

[b95] Uversky V. N. The mysterious unfoldome: structureless, underappreciated, yet vital part of any given proteome. J Biomed Biotechnol 2010, 568068; 10.1155/2010/568068 (2010). 20011072 PMC2789583

[b96] Ward J. J., Sodhi J. S., McGuffin L. J., Buxton B. F. & Jones D. T. Prediction and functional analysis of native disorder in proteins from the three kingdoms of life. J Mol Biol 337(3), 635–645 (2004). 15019783 10.1016/j.jmb.2004.02.002

[b97] Iakoucheva L. M., Brown C. J., Lawson J. D., Obradovic Z. & Dunker A. K. Intrinsic disorder in cell-signaling and cancer-associated proteins. J Mol Biol 323(3), 573–584 (2002). 12381310 10.1016/s0022-2836(02)00969-5

[b98] Iakoucheva L. M. *et al.* The importance of intrinsic disorder for protein phosphorylation. Nucleic Acids Res 32(3), 1037–1049 (2004). 14960716 10.1093/nar/gkh253 PMC373391

[b99] Yang X. J. Multisite protein modification and intramolecular signaling. Oncogene 24(10), 1653–1662 (2005). 15744326 10.1038/sj.onc.1208173

[b100] Uversky V. N. Intrinsic disorder-based protein interactions and their modulators. Curr Pharm Des 19(23), 4191–4213 (2013). 23170892 10.2174/1381612811319230005

[b101] Pejaver V. *et al.* The structural and functional signatures of proteins that undergo multiple events of post-translational modification. Protein Sci 23(8), 1077–1093 (2014). 24888500 10.1002/pro.2494 PMC4116656

[b102] Varewijck A. J. & Janssen J. A. Insulin and its analogues and their affinities for the IGF1 receptor. Endocr Relat Cancer 19(5), F63–75 (2012). 22420005 10.1530/ERC-12-0026

[b103] Slieker L. J. *et al.* Modifications in the B10 and B26-30 regions of the B chain of human insulin alter affinity for the human IGF-I receptor more than for the insulin receptor. Diabetologia 40 Suppl 2, S54–61 (1997). 9248702 10.1007/s001250051402

[b104] Minde D. P., Halff E. F. & Tans S. Designing disorder: Tales of the unexpected tails. Intrinsically Disord Proteins 1, e26790; 10.4161/idp.26790 (2013). PMC5424805 28516025

[b105] Uversky V. N. Proteins without unique 3D structures: biotechnological applications of intrinsically unstable/disordered proteins. Biotechnol J 10(3), 356–366 (2015). 25287424 10.1002/biot.201400374

[b106] Santner A. A. *et al.* Sweeping away protein aggregation with entropic bristles: intrinsically disordered protein fusions enhance soluble expression. Biochemistry 51(37), 7250–7262 (2012). 22924672 10.1021/bi300653m PMC4141500

[b107] Meyer D. E., Trabbic-Carlson K. & Chilkoti A. Protein purification by fusion with an environmentally responsive elastin-like polypeptide: effect of polypeptide length on the purification of thioredoxin. Biotechnol Prog 17(4), 720–728 (2001). 11485434 10.1021/bp010049o

[b108] Banki M. R., Feng L. & Wood D. W. Simple bioseparations using self-cleaving elastin-like polypeptide tags. Nat Methods 2(9), 659–661 (2005). 16074986 10.1038/nmeth787

[b109] Bellucci J. J., Amiram M., Bhattacharyya J., McCafferty D. & Chilkoti A. Three-in-one chromatography-free purification, tag removal, and site-specific modification of recombinant fusion proteins using sortase A and elastin-like polypeptides. Angew Chem Int Ed Engl 52(13), 3703–3708 (2013). 23424160 10.1002/anie.201208292 PMC3723126

[b110] Obradovic Z., Peng K., Vucetic S., Radivojac P. & Dunker A. K. Exploiting heterogeneous sequence properties improves prediction of protein disorder. Proteins 61 Suppl 7, 176–182 (2005). 16187360 10.1002/prot.20735

